# Long-Term Severe In Vitro Hypoxia Exposure Enhances the Vascularization Potential of Human Adipose Tissue-Derived Stromal Vascular Fraction Cell Engineered Tissues

**DOI:** 10.3390/ijms22157920

**Published:** 2021-07-24

**Authors:** Myroslava Mytsyk, Giulia Cerino, Gregory Reid, Laia Gili Sole, Friedrich S. Eckstein, David Santer, Anna Marsano

**Affiliations:** 1 Department of Cardiac Surgery, University Hospital Basel, 4031 Basel, Switzerland; myroslava.mytsyk@gmail.com (M.M.); cerinoa.giulia@gmail.com (G.C.); gregory.reid@usb.ch (G.R.); laia.gilisole@unibas.ch (L.G.S.); Friedrich.eckstein@usb.ch (F.S.E.); david.santer@usb.ch (D.S.); 2 Department of Biomedicine, University Basel, 4031 Basel, Switzerland

**Keywords:** hypoxic culture, stromal vascular fraction cells, engineered tissues, in vivo angiogenesis

## Abstract

The therapeutic potential of mesenchymal stromal/stem cells (MSC) for treating cardiac ischemia strongly depends on their paracrine-mediated effects and their engraftment capacity in a hostile environment such as the infarcted myocardium. Adipose tissue-derived stromal vascular fraction (SVF) cells are a mixed population composed mainly of MSC and vascular cells, well known for their high angiogenic potential. A previous study showed that the angiogenic potential of SVF cells was further increased following their in vitro organization in an engineered tissue (patch) after perfusion-based bioreactor culture. This study aimed to investigate the possible changes in the cellular SVF composition, in vivo angiogenic potential, as well as engraftment capability upon in vitro culture in harsh hypoxia conditions. This mimics the possible delayed vascularization of the patch upon implantation in a low perfused myocardium. To this purpose, human SVF cells were seeded on a collagen sponge, cultured for 5 days in a perfusion-based bioreactor under normoxia or hypoxia (21% and <1% of oxygen tension, respectively) and subcutaneously implanted in nude rats for 3 and 28 days. Compared to ambient condition culture, hypoxic tension did not alter the SVF composition in vitro, showing similar numbers of MSC as well as endothelial and mural cells. Nevertheless, in vitro hypoxic culture significantly increased the release of vascular endothelial growth factor (*p* < 0.001) and the number of proliferating cells (*p* < 0.00001). Moreover, compared to ambient oxygen culture, exposure to hypoxia significantly enhanced the vessel length density in the engineered tissues following 28 days of implantation. The number of human cells and human proliferating cells in hypoxia-cultured constructs was also significantly increased after 3 and 28 days in vivo, compared to normoxia. These findings show that a possible in vivo delay in oxygen supply might not impair the vascularization potential of SVF- patches, which qualifies them for evaluation in a myocardial ischemia model.

## 1. Introduction

Following myocardial infarction (MI), one of the first treatments is the restoration of the macrocirculation (e.g., by a coronary artery bypass graft (CABG)). The microcirculation however, is often also compromised by the presence of a rarefied capillary network in the areas of the infarcted myocardium [1] not restored by standard clinical revascularization interventions. Therefore, there is still an unmet clinical need for the development of an additional angiogenic therapy targeting the induction of new capillaries for the treatment of subacute or chronic cardiac ischemic disease. In the past decades, pre-clinical and clinical trials have investigated the therapeutical potential of adult mesenchymal stem/stromal cell (MSC)-based treatments [2,3,4]. Although the application is safe [5,6], the long-term results are still unclear or not yet available [7]. Nevertheless, existing trials were able to show that the delivery of MSC during CABG might improve cardiac function in non-revascularized tissue [6,8]. The mechanisms underlying the benefits of MSC-based therapy are still yet to be elucidated. However, animal studies of subacute/chronic cardiac ischemia have demonstrated that the observed benefits of MSC-based treatments in promoting angiogenesis and cardiomyocyte contractility largely depend on the effect of their secretome (e.g., release of angiogenic, pro-survival factors) [9,10].

Among the several promising types of adult MSC investigated so far, stromal vascular fraction (SVF) cells stand out for their high therapeutical potential in the context of cardiac ischemia thanks to their capacity to promote vascularization and cardiac functionality [11], as well as their intrinsic cardioprotective effect [12]. Contrary to MSC from other tissues, SVF cells are a mixed cell population rich in cells from the vascular component, such as endothelial cells and pericytes, besides MSC and hematopoietic cells. Our approach is to induce angiogenesis in a chronic ischemic heart, based on using an epicardial-implanted SVF cell-based engineered tissue functioning as a delivery system of angiogenic and pro-survival factors. To ensure a sustained release of factors in vivo, it is essential that the engineered patches are promptly vascularized upon implantation.

One limiting factor that could drastically reduce the therapeutic efficacy of MSC or SVF cell-based approaches is the low in vivo cell engraftment due to the high cell death during the delivery, together with a difficult homing process. Cell engraftment in a harsh microenvironment such as that of the ischemic myocardium can be really challenging. It has been demonstrated that the delivery of cells by means of a scaffold or as cell sheets could improve cell survival compared to other methods such as cell intra-myocardial injection [13]. Previous results showed that further in vitro pre-organization of the cells in engineered tissues by using a direct perfusion of SVF cells on three-dimensional (3D) collagen-based scaffold strongly enhances the survival of the vascular cell component and the release of angiogenic factors in vitro, as well as their vascularization capability in a subcutaneous rat model [14].

Although the vascularization of the patches engineered under perfusion was accelerated at early time points compared to a simple static culture, up to 7 days in vivo implantation were necessary to reach a uniform infiltration of blood vessels throughout the whole construct in a model of rat subcutaneous pocket [14]. Considering the significant long time needed for the in vivo vascularization of mm-thick constructs, SVF-patches might be exposed to hypoxia, which might affect the SVF cell composition and secreted factors. Short preconditioning of MSC by hypoxia increases the production of angiogenic and pro-survival factors [15,16], and therefore, their angiogenic potential [17,18]. Moreover, while an acute short term oxygen depletion provokes cell damage leading to apoptosis, prolonged exposure to hypoxia is known to induce somatic cells to adapt to the new microenvironmental condition and to release cardioprotective factors [19,20].

Therefore, in this study we aimed to investigate the possible changes in the in vitro SVF cell composition as well as the in vivo angiogenic potential of an SVF-based engineered patch cultured for 5 days under severe hypoxia. In particular, we hypothesized that the hypoxic culture of the SVF-based patches promotes the vascularization of the engineered tissue and the cell engraftment upon in vivo implantation in a rat subcutaneous model.

## 2. Results

### 2.1. Static vs. Dynamic Culture Conditions

**Figure 1:**
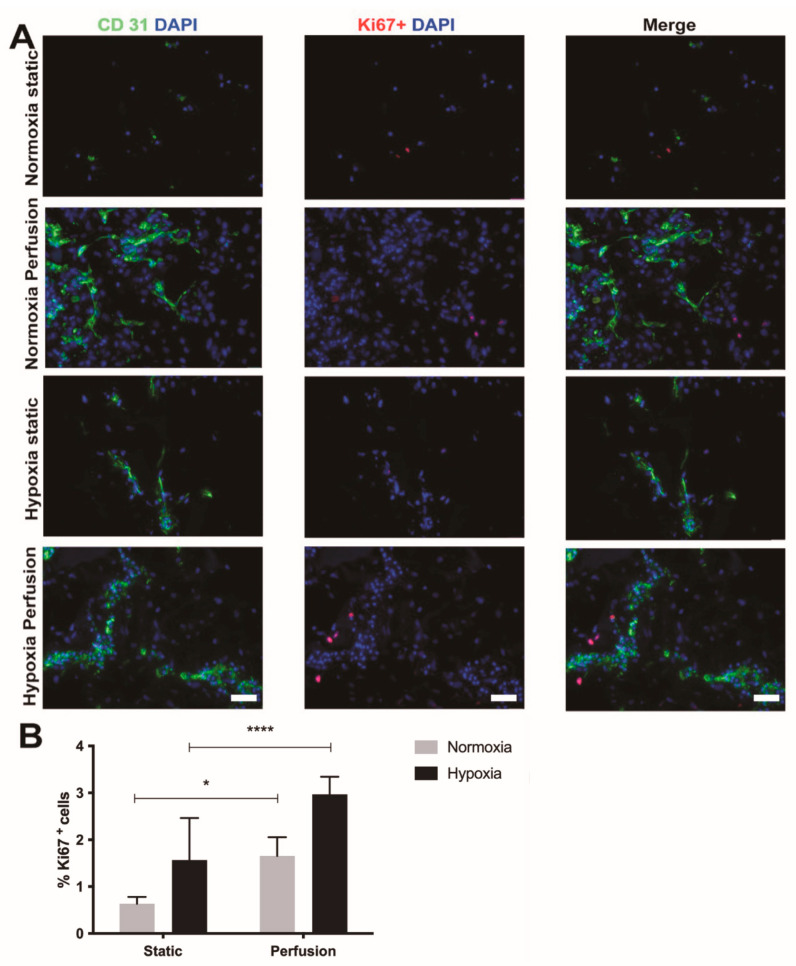
Stromal vascular fraction (SVF) cell-based constructs cultured in perfusion or static condition. (**A**) Immunostaining for endothelial cell marker PECAM-1 (CD31, green), proliferating cell marker Ki67 (red) of SVF cells cultured either in static or perfusion condition at either 21% or <1% O_2_. Cell nuclei were stained with DAPI (blue). Scale bar = 75 µm. (**B**) Percentage of proliferating cells for static and perfusion conditions cultured under in vitro normoxia and hypoxia. *n* = 12 (1 donor). **** *p* < 0.0001. * *p* = 0.049.

First, we compared the SVF cell proliferation and endothelial cell organization following the culture in static or dynamic condition at either in vitro normoxia or severe hypoxia. Dynamic culture promoted endothelial cell elongation in both hypoxia and normoxia. Contrary to dynamic culture, the static culture did induce the formation of elongated endothelial structures, only under severe hypoxia condition (Figure 1A).

Although the number of dividing cells was quite low overall, severe hypoxia exposure enhanced cell proliferation in both dynamic and static conditions (Figure 1A,B). After five days, significantly more Ki67-positive cells were indeed quantified both under hypoxic (*p* < 0.0001) as well as normoxic (*p* = 0.049) conditions in the dynamic culture compared to the static culture. (Figure 1B).

### 2.2. Hypoxia vs. Normoxia in Dynamic Culture Conditions

Compared to the static condition, dynamic culture was confirmed to lead to a superior endothelial cell elongation and cell proliferation not only in normoxia, as previously shown [14], but also under hypoxic culture. Based on these findings, the following experiments included only engineered tissues generated in perfusion-based bioreactors.

**Figure 2:**
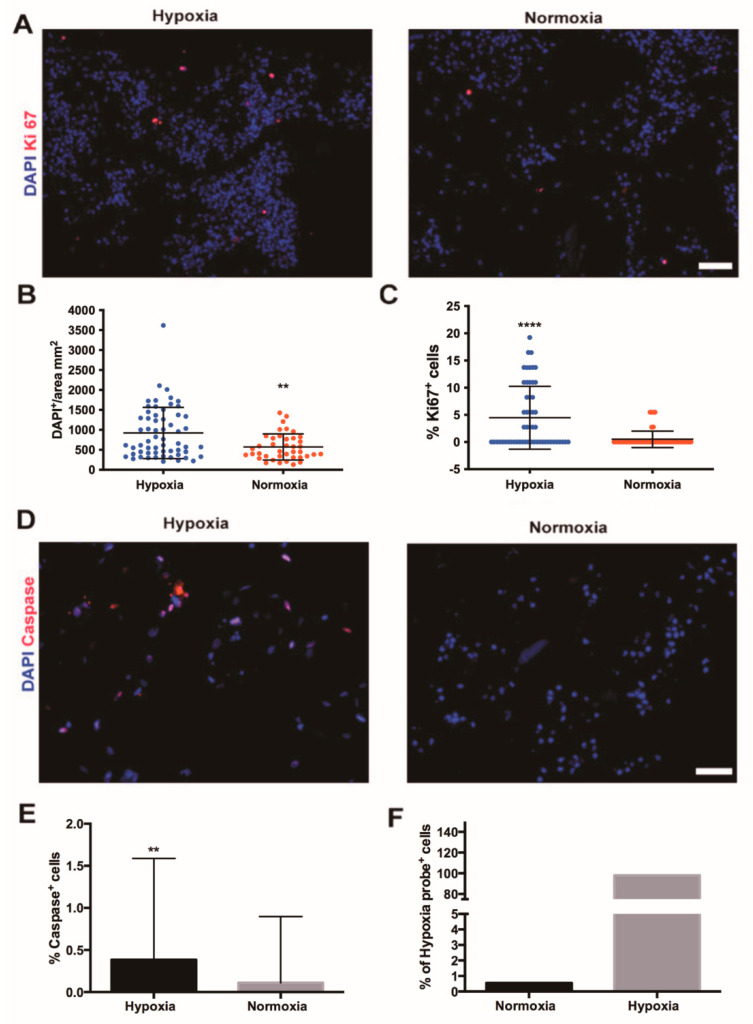
Effects of perfusion culture under hypoxia or normoxia on human stromal vascular fraction cell proliferation and apoptosis. (**A**) Immunofluorescence staining for Ki67, the marker of proliferation (red) of perfused SVF cell-based constructs in either hypoxia or in vitro normoxia. Cell nuclei were stained with DAPI (blue). Scale bar = 70 µm. Quantification of total number of SVF cells (DAPI-positive cells) (**B**) and percentage of proliferating (Ki67-positive) cells (**C**) in constructs cultured in perfusion culture under high or low oxygen tension. **** *p* < 0.00001; ** *p* < 0.001. *n* = 72 (6 donors). (**D**) Immunofluorescence staining for marker of apoptosis (cleaved caspase-3, red) of SVF cell-based constructs cultured in hypoxia and in vitro normoxia. Cell nuclei were stained with DAPI (blue). Scale bar = 35 µm. (**E**) Percentage of apoptotic cells, positive for cleaved caspase-3 in constructs cultured in perfusion culture under high or low oxygen tension. ** *p* < 0.001. *n* = 36 (3 donors). (**F**) Flow cytometry analysis of SVF cells cultured in perfusion in normoxia or hypoxia positive to the pimonidazole. Donor *n* = 1. Comparison was performed with non-parametric test (Mann–Whitney-U).

After five days of perfusion culture, SVF cells formed large cell aggregates under both hypoxia and normoxia (Figure 2A). To confirm the nuclear expression of Ki67, phosphohistone H3 staining was performed (Supplementary Materials, Figure S1), which showed proliferating cells only in the hypoxia-treated patches compared to those cultures under normal oxygen tension. Compared to normoxia, hypoxia markedly increased the total number of cells as well as the count of the proliferating (Ki67-positive) cells (both *p* < 0.0001; Figure 2B,C). The number of apoptotic cells, positively stained for cleaved caspase-3, was very low in both conditions (Figure 2D,E). Nevertheless, a significant, superior number of apoptotic cells under hypoxia (*p* < 0.001; Figure 2E) was observed. No difference between the center or the edges of the engineered tissues was noticed. Flow cytometry analysis performed on live cells retrieved from the engineered tissue after five days of culture showed that more than 98% of the cells were positive to the hypoxia-probe (pimonidazole HCl) following the exposure to hypoxic conditions. Instead, in in vitro normoxia less than 1% of cells were positive to the hypoxia-probe (Figure 2F).

**Figure 3:**
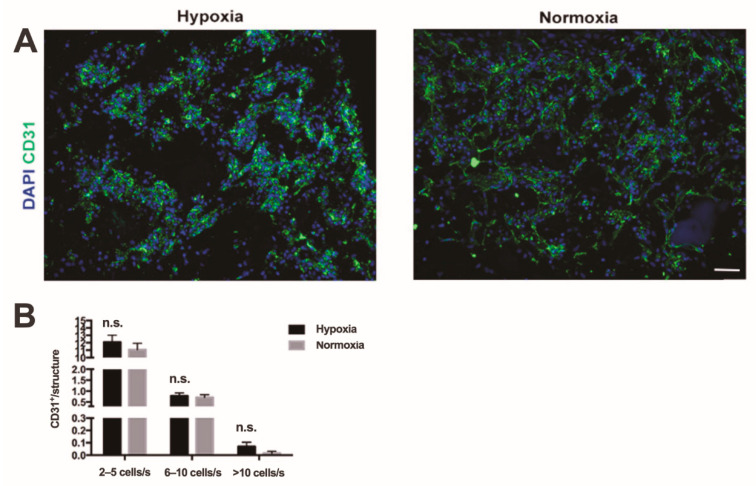
In vitro endothelial cell organization. (**A**) Immunofluorescence staining endothelial cell marker (CD31, green) of constructs with SVF cells cultured in hypoxia and in normoxic culture. Cell nuclei were stained with DAPI (blue). Scale bar = 70 µm. (**B**) Quantification of number of organized endothelial cells in small (3–5 cells per structure), middle-size (5–10 cells per structure), and complex (over 10 cells per structure) cord-like structures. n.s. = not statistically significant; s: structure; *n* = 72 (6 donors). Comparison was performed with non-parametric test (Mann–Whitney-U).

Endothelial cells formed large aggregates or elongated vessel-like structures during perfusion culture (Figure 3A). The total number of CD31 positive cells that showed an organized cord-like structure were quantified and divided based on the number of cells inside of each structure. Cord-like structures formed by only two elongated endothelial cells were not considered. There was no significant difference in the number of cord-like structures formed under hypoxia compared to normoxia. However, there was a tendency for a higher number of complex cord-like structures when the number of participating endothelial cells was over 10 (n.s., Figure 3B).

**Figure 4:**
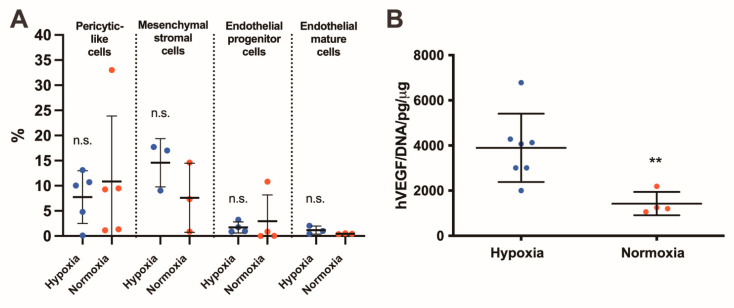
In vitro characterization of stromal vascular fraction (SVF)-based constructs. SVF composition and angiogenic factor release. (**A**) Flow cytometry analysis: percentage of different SVF subpopulations, namely, pericytic-like (CD45^−^, CD34^−^, CD146^+^), mesenchymal stromal cells (MSC, CD45^−^, CD90^+^, CD73^+^), and endothelial progenitor (CD45^−^, CD31^+^, CD34^+^) and mature (CD45^−^, CD31^+^, VEGFR2^+^) cells, following 3D perfusion-based culture in in vitro normoxia or hypoxia. Values are presented as percentage of living cells. n.s. = not statistically significant. *n* = 5. (**B**) Quantification of human VEGF released in the supernatant by SVF cultured under severe hypoxia or normoxia culture normalized by the total DNA content. ** *p* < 0.001. *n* = 4. hVEGF: human vascular endothelial growth factor; DNA: desoxyribonucleic acid. Comparison was performed with non-parametric test (Mann–Whitney-U).

Perfusion culture stimulates the pericyte subpopulation of SVF cells, mesenchymal cell subpopulation, endothelial progenitor and mature cells. No significant difference was found between the culture under in vitro normoxia or severe hypoxia (Figure 4A). In hypoxia and normoxia, the increase in relation to the initial population was similar in all subpopulations (pericyte-like cells: 1.9 ± 2.4 vs. 1.7 ± 1.2-fold increase, *p* = 0.89; MSC 8.3 ± 6.6 vs. 4.1 ± 6.1-fold increase, *p* = 0.40; endothelial progenitor cells: 1.4 ± 1.1 vs. 1.5 ± 2.7-fold increase, *p* = 0.49; mature cells: 12.1 ± 9.0 vs. 4.9 ± 2.0-fold increase, *p* = 0.10). In hypoxia culture, the SVF releasing profile of human vascular endothelial growth factor (VEGF) increased significantly in ELISA assay (*p* < 0.001; Figure 4B).

### 2.3. In Vivo Vascularization and Human Cell Engraftment

**Figure 5:**
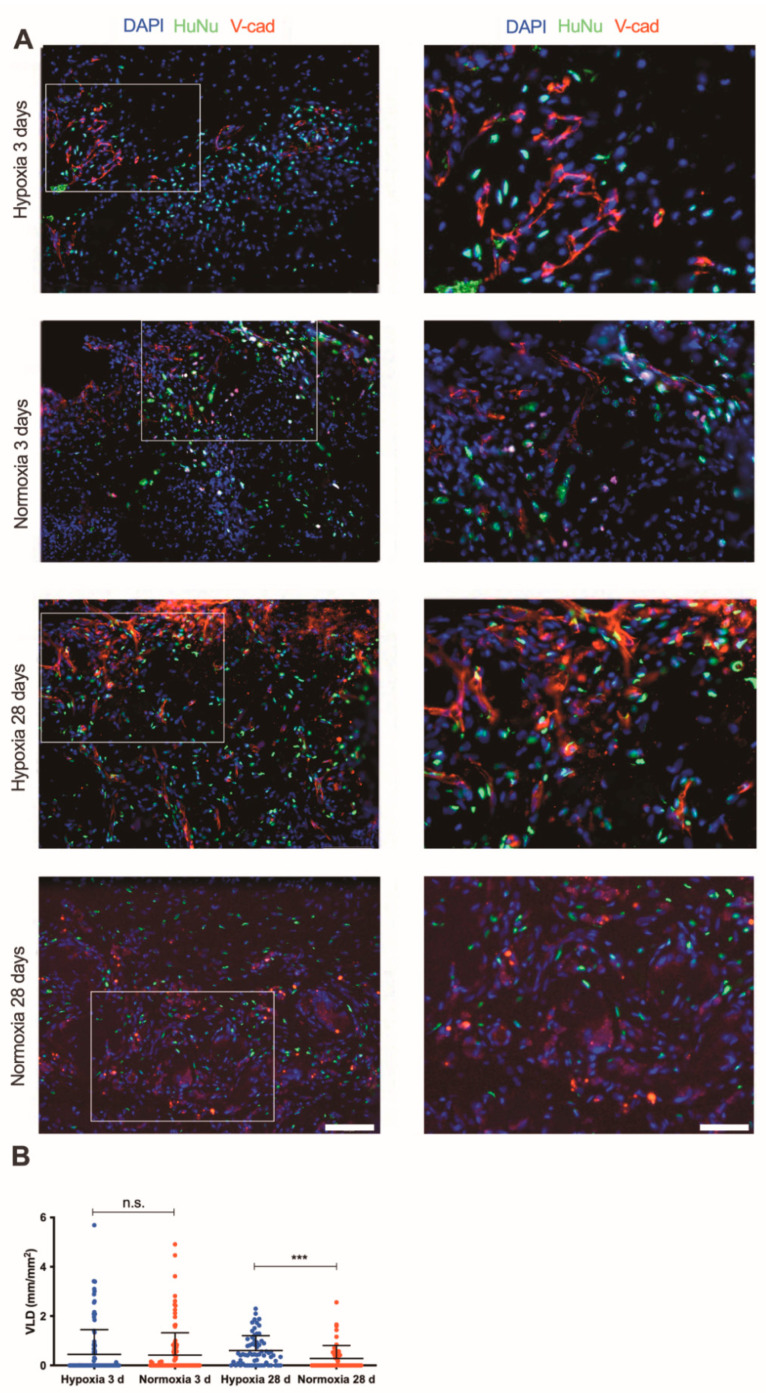
In vivo vascularization potential of SVF-based patches. (**A**) Immunofluorescence staining endothelial cells (Ve-caderin, red), human nuclei specific marker (HuNu, green) of constructs generated by SVF cells in severe hypoxia and normoxia perfusion-based culture with investigation time points 3 and 28 days (d) at low (20×) and high (40×) magnifications (scale bars = 50 µm and 25 µm, respectively). (**B**) Quantification of vessel length density (VLD) of constructs cultured under severe hypoxia or in vitro normoxia after implantation in vivo with investigation time points 3 and 28 days. *** *p* < 0.001; n.s. = not statistically significant. Comparison was performed with one-way ANOVA. Number of donors: 3 donors (at least duplicate biological samples per donor).

The in vivo vascularization potential and the cell engraftment of the engineered tissues were assessed in subcutaneous pockets in a nude rat model. After three days, SVF cell-based patches cultured under hypoxia did not show superior vessel ingrowth as compared to constructs cultured under normoxic condition (Figure 5A,B). The vessel ingrowth for both conditions was mainly limited at the border of the implant with the host tissue. After 28 days, constructs cultured in hypoxia were completely invaded by host vascularization. Indeed, a negligible number of human cells were found to be part of the newly generated blood vessels (Figure 5A). Vessels infiltrated from the host surrounding tissue and reached the center of the constructs. The vessel length density quantification showed that hypoxia-cultured SVF cells significantly enhanced the vessel ingrowth within the implants compared to normoxia after 28 days (*p* < 0.001; Figure 5B). Further immunofluorescence staining depicted the blood vessel morphology and showed the formation of arterioles surrounded by smooth muscle cells (positive for alpha-SMA staining) as well as stable capillary structures covered by pericytes (positive for NG2 staining) (Figure S2).

**Figure 6:**
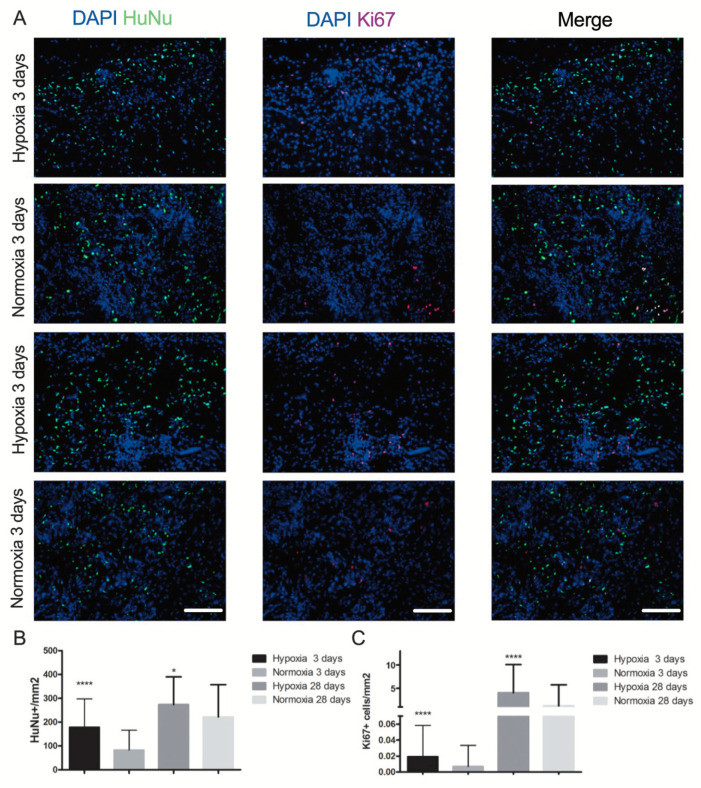
In vivo human cell engraftment and proliferation. (**A**) Representative images of implanted construct cultured in hypoxia or normoxia culture. Immunofluorescence staining for specific human nuclei antibody HuNu (green) and for the proliferation marker Ki67 (red) at time points 3 and 28 days (d). Cell nuclei were stained by DAPI (blue). Scale bar = 40 µm. (**B**) Quantification of total number of implanted human cells (double positive for HuNu and DAPI) and (**C**) proliferation cells (triple positive for HuNu, Ki67 and DAPI) following 3 and 28 days in vivo implantation. * *p* < 0.01, **** *p* < 0.00001. Number of donors: 3 (at least duplicate biological samples per donor). Comparison was performed with one-way ANOVA.

In both normoxic and hypoxic conditions, human cells were present quite uniformly throughout the whole implanted constructs at either 3 or 28 days (Figure 6A). Nevertheless, SVF-based patches cultured in hypoxia conditions had a significantly higher number of human cells following either 3 or 28 days of in vivo implantation compared to normoxia culture constructs (Figure 6B). A very low number of proliferative cells (double positive for ki67 and DAPI) was observed overall in all the in vivo engineered tissues (Figure 6A). However, the number of proliferating cells in patches cultured in hypoxia was significantly higher compared to normoxic condition at both the in vivo time points (both *p* < 0.0001; Figure 6C).

## 3. Discussion

Our findings showed that compared to the culture in normoxic condition, in vitro long-term exposure to hypoxia of SVF-based engineered tissues did not change the main subpopulation composition. However, hypoxia conditioning enhanced the in vitro release of VEGF, as well as their in vivo vascularization capacity and cell engraftment.

This study showed a superior vessel length density as well as a higher percentage of engrafted human cells in SVF-patches cultured under severe hypoxia compared to normoxia following 28 days of culture. Nevertheless, a very limited number of human cell origin were observed in the newly generated blood vessels. A limitation of this study was that an assessment of the functional perfusion of the blood vessels at the moment of the animal sacrifice was not performed. However, the morphological analysis of the blood vessels in both experimental conditions revealed the presence of arterioles and stable capillaries covered by pericytes, as it was described previously [14].

In this study, for the first time, the combined effect of a long-term (5 days) and very low oxygen tension (below 1%—severe condition) was investigated in a perfused-based culture system of several mm-thick engineered constructs. The direct perfusion of low-oxygenated culture medium through the pores of the 3D engineered tissues was chosen to promote a uniform exposure of all the cells to a uniform microenvironmental condition. Indeed, in static culture conditions, gradients of oxygen due to the oxygen diffusion limit (circa 200 µm) [21] would create an inconsistent microenvironment.

Our ultimate goal instead is to mimic in vitro a possible delay in the vascularization process of a mm-thick engineered tissues, simulating the situation of patch implantation in a low perfused area of an ischemic myocardium. The model proposed here mimics, in part, a single aspect of an ischemic cardiac tissue, in which the cells are not only exposed to a low oxygen tension, but also to a lack of nutrients supply and to necrotic, inflammatory as well as ventricle remodeling processes.

Although the ischemic heart rat model might represent a more clinically-relevant approach, in this study we chose subcutaneous implantation in nude rats, as it is the most efficacious and yet simple model to assess the intrinsic vascularization capacity of engineered tissues, removing any other confounding cellular process related to, e.g., inflammation, apoptosis and fibrosis [22].

Previously published results have focused on the paracrine effects of medium conditioned by SVF-patches cultured in severe hypoxia on a two-dimensional (2D) in vitro cardiac model of dysfunctional cardiomyocytes [23]. Compared to static culture, the secretome released by hypoxia cultured SVF-based patches was indeed enriched with pro-survival factors (such as the hepatocyte growth factor and the insulin growth factor), and therefore, capable of partially restoring the contractility of dysfunctional cardiomyocytes.

Although a statistically significant difference was found in cell proliferation and apoptosis between hypoxia and normoxia conditions, the actual numbers of dividing and apoptotic cells were extremely low. This fact could be related to the time frame of hypoxia exposure and the different level of oxygen to those adopted in other studies [24].

A superior percentage of proliferating cells in hypoxia cultured patches was confirmed by using both Ki67 and phospho-histone H3 (pH3) antibodies following 5 days of in vitro culture in perfusion. In particular, a higher difference between normoxia and hypoxia cultured patches was observed in the pH3 expression compared to Ki67, possibly due to the fact that Ki67 is present in all phases of the cell cycle (except for quiescent phase, the G0) while pH3 is specific for cells only in late G2 and mitosis phases [25].

The in vitro and in vivo findings showed quite high variability, which could be mainly due to high inter-donor variability.

Other studies confirmed our findings that the culture at very low hypoxic oxygen tension (<0.1% oxygen) increases the production of angiogenic factors, such as VEGF-A in human adipose-derived stem cells [9]. Furthermore, under prolonged hypoxic culture (<1% of O_2_ during 4–13 days) angiogenic potential of human adipose-derived stem cells was improved as shown by an increase in released VEGF [26], even up to levels similar to those achieved with the addition of exogenous angiogenic growth factors [27].

## 4. Materials and Methods

### 4.1. Stromal Vascular Fraction Cell Isolation

Human abdomen adipose tissue or lipoaspirate were obtained with written informed consent from individuals undergoing plastic surgery according to a protocol approved by the Ethical Committee of the University Hospital Basel.

The adipose tissue was minced (these steps were not performed in case of lipoaspirates) before proceeding with the digestion with 0.075% *w*/*v* type II collagenase solution (Worthington Biochemical Corporation, Lakewood, NJ, USA) at 37 °C for 60 min under continuous shaking. After 10 min of centrifugation at 1.500 rpm and removal of floating adipocytes, the remaining cell pellet was washed with high glucose Dulbecco’s Modified Eagle Medium (DMEM, Thermo Fisher Scientific Inc., Waltham, MA, USA) supplemented with 10% fetal bovine serum (FBS) (HyClone, Thermo Fisher Scientific Inc., Waltham, MA, USA) and filtered sequentially through a 100 and 70 µm nylon-mesh strainer. Freshly isolated SVF cells were frozen and stored in liquid nitrogen [28].

### 4.2. Three Dimensional Perfusion-Based Culture

Stromal vascular fraction cells were seeded right after thawing on collagen discs (diameter: 8, thickness: 3 mm, Avitene Ultrafoam^TM^, BD Biosciences, Allschwil, Switzerland) in perfusion-based bioreactors by using a 3.0 mL/min bi-directional flow (PHD ULTRATM 2000, Harvard Apparatus, Holliston, MA, USA) for 24 h. Cell-based collagen sponges were then further cultured in a tri-gas humidified incubator (Thermo Fisher Scientific Inc., Waltham, MA, USA) at 5% CO_2_ and 37 °C under uni-directional flow (Ismatec Reglo Digital MS-4/8, Cole-Parmer GmbH, Wertheim, Germany) for 5 days with at a flow rate of 3 mL/min at either 21% (in vitro normoxia) or <1% (up to 0.2%, severe hypoxia) of oxygen. Culture medium consisted of DMEM high glucose (4500 mg/L, Thermo Fisher Scientific Inc., Waltham, MA, USA) supplemented with 10% (*v*/*v*) FBS, 1% (*v*/*v*) penicillin/streptomycin and 1% (*v*/*v*) glutamine (all Thermo Fisher Scientific Inc., Waltham, MA, USA).

### 4.3. In Vivo Subcutaneous Implantation in Nude Rats

Animals were treated in accordance with the Swiss Federal guidelines for animal welfare, after approval from the Veterinary Office of the Canton of Basel-Stadt (approval number: 23511-2608, approval date: 28 May 2013, Basel, Switzerland). The SVF-based engineered tissues were implanted ectopically in 8 weeks-old male nude rats (rat weight circa 250 g, Harlan Laboratories, Horst, The Netherlands). Animals were anesthetized using isoflurane (1.5–3% (*v*/*v*)) in 0.6 L/min oxygen. Four constructs were implanted per rat in four independent subcutaneous pockets formed in the back of the animal. Buprenorphin (0.05 mg/kg/dose) was administered for the first time 1 h before surgery and then every 12 h for 3 days. The animals were sacrificed after 3 or 28 days after implantation.

### 4.4. In Vitro Hypoxia Condition Assessment

After 5 days of in vitro culture, engineered tissues were perfused with 0.1 mM pimonidazole hydrochloride (Hypoxyprobe Inc., Burlington, MA, USA) in the bioreactors for 2 h. Samples were then digested in a 0.15 (*w*/*v*) % type II collagenase (Worthington Biochemical Corporation, Lakewood, NJ, USA) in PBS solution for 10 min at 37 °C and the released cells were incubated first with anti-pimonidazole primary antibody (1:125, mouse IgG1, Hypoxyprobe Inc., Burlington, MA, USA) for 1 h and then with fluorescein isothiocyanate (FITC)-conjugated secondary antibody for 1 h (1:100, mouse IgG1). Stained cells were then analyzed by using the flow cytometry (LSRFortessa™, BD Biosciences, Allschwil, Switzerland).

### 4.5. Flow Cytometry

The in vitro samples were digested in 0.15 (*v*/*v*) % collagenase at 37 °C, 2/3 of the released cells were incubated with CD45 BV605, CD34 APC-Cy7, CD146 PE, CD73 APC, CD90 FITC, VEGFR2 PE and PECAM-1 (CD31) FITC antibodies for 30 min on ice. The other 1/3 isolated cells were used as control (unstained). Analysis was performed with the LSRFortessa™ flow cytometer (BD Biosciences, Allschwil, Switzerland). Stromal vascular fraction subpopulations were analyzed and identified as follows: pericytes (CD45^−^, CD34^−^, CD146^+^); MSC (CD45^−^, CD73^+^, CD90^+^); endothelial progenitor cells (CD45^−^, CD31^+^, CD34^+^); endothelial mature cells (CD45^−^, CD31^+^, VEGFR2^+^). All values have been normalized to the initial SVF composition. Results were analyzed with FlowJo (v10, Becton, Dickinson and Company, Franklin Lakes, NJ, USA, Single cells analysis).

### 4.6. Histology

In vitro and in vivo constructs were fixed with 1% (*w*/*v*) PFA for 2 h, followed by 25% (*w*/*v*) sucrose incubation for 12 h. Samples were then embedded in optimal cutting temperature (OCT) compound (CellPath, Newton, UK), frozen in freezing isopentane, and cryosectioned (12 μm thickness). Cryosections were incubated for 1 h in 0.3% *w*/*v* Triton X-100 and 2% *v*/*v* normal goat serum, or 5% *v*/*v* donkey serum in PBS (blocking buffer), and then for 1 h in the following primary antibodies: mouse anti-PECAM-1 (CD31, endothelial cells, 1:100, LubioScience Gmbh, Zürich, Switzerland), rabbit anti-Ki67 (proliferating cells, 1:100, Abcam, Cambridge, UK), rabbit anti-phospho-histone H3 (Ser28) (1:400, Cell Signaling Technology, Danvers, MA, USA) and rabbit anti-cleaved caspase3 (cells in apoptosis, 1:100, Cell Signaling, Technology Inc., Danvers, MA, USA), mouse monoclonal anti-Human Nuclei (HuNu) (clone 235-1, human cells, 1:100, Merck Millipore, Burlington, MA, USA), and goat anti-Ve-Cadherin (endothelial cells, 1:200, Santa Cruz Biotechnology, Dallas, TX, USA). Tissue sections were then incubated in the dark for 1 h in fluorescently labeled Alexa488 and Alexa546 secondary antibody (dilution 1:200, Thermo Fisher Scientific Inc., Waltham, MA, USA). Cell nuclei were stained with 4′,6-Diamidine-2′-phenylindole dihydrochloride (DAPI, 300nM, Thermo Fisher Scientific Inc., Waltham, MA, USA).

### 4.7. Image Analysis

Analysis was performed on images acquired with a 20× objective by using a fluorescent microscope (BX63) with a digital camera (DP80, both Olympus Corporation, Tokyo, Japan). At least 6 cross-sections were analyzed with 10 images per sample per duplicates per 6 donors (in vitro) per 3 donors (in vivo) per experimental group.

In vitro cell density was calculated as number of nuclei (DAPI-positive cells) normalized to the analyzed field area. The vessel-like organization was assessed as the percentage of aligned and elongated cord-structures composed by 2–5, 6–10 or >10 endothelial cells (CD31-positive cells).

Vessel length density (VLD) was calculated for in vivo samples as the sum of all the vessel length normalized to the analyzed field area. The total vessel length was assessed by tracing the CD31-positive blood vessels by using CellSense software (Olympus Corporation, Tokyo, Japan).

### 4.8. Human VEGF Release Quantification

Amount of human vascular endothelial growth factor (VEGF was quantified after 5 days of in vitro culture by enzyme-linked immunosorbent assay (ELISA, R&D Systems Inc., MN, USA) analysis and normalized to the amount of DNA present in each construct. Total amount of DNA present in each sample was analyzed by a CyQuant kit (Thermo Fisher Scientific Inc., Waltham, MA, USA) as described in the technical sheet.

### 4.9. Statistical Analysis

Statistical analysis was performed with Prism 9.1.0 software (GraphPad Software, San Diego, USA). All data are expressed as mean ± standard deviation. The figure legends of all figures include the type of statistical test used. Differences were considered statistically significant when *p* < 0.05.

## 5. Conclusions

Three-dimensional-SVF patches need prompt vascularization upon in vivo implantation to maintain viability, and therefore, their therapeutical potential. However, the patch implantation in tissues with a chronic lack of oxygen might present a challenge. We showed that exposure to severe and prolonged hypoxia conditions in vitro did not compromise the angiogenic potential or the viability of the SVF-patches in vivo, but it further enhanced these effects. These findings show that SVF-patches might be a good angiogenic treatment option for ischemic tissues (e.g., skeletal or cardiac muscles). Furthermore, the investigated hypoxia experimental condition could even be suggested as a possible additional in vitro manipulation to further enhance their therapeutic efficacy.

## Data Availability

Not applicable.

## Supplementary Materials

The following are available online at https://www.mdpi.com/article/10.3390/ijms22157920/s1.

## Abbreviations

APC	Allophycocyanin
BV605	Brilliant Violet 605
CD	Cluster of Differentiation
Cy7	Cyanine 7
DAPI	4′,6-Diamidine-2′-phenylindole dihydrochloride
DMEM	Dulbecco’s Modified Eagle Medium
ELISA	Enzyme-Linked Immunosorbent Assay
FBS	Fetal Bovine Serum
FITC	Fluorescein Isothiocyanate
HuNu	Human Nuclei
MSC	Mesenchymal Stem/stromal Cells
OCT	Optimal Cutting Temperature
PE	Phycoerythrin
PECAM-1	Platelet Endothelial Cell Adhesion Molecule (PECAM-1) also known as cluster of differentiation 31 (CD31)
pH3	phospho-histone H3
SVF	Stromal Vascular Fraction
VEGF	Vascular Endothelial Growth Factor
VEGFR2	Vascular Endothelial Growth Factor Receptor 2
VLD	Vessel Length Density
*v*/*v*	Volume/Volume
*w*/*v*	Weight/Volume
