# Supplementary material for: Long-Term Severe In Vitro Hypoxia Exposure Enhances the Vascularization Potential of Human Adipose Tissue-Derived Stromal Vascular Fraction Cell Engineered Tissues
Source: Int J Mol Sci. 2021 Jul 24;22(15):7920. doi: 10.3390/ijms22157920 (PMC8348696; doi:10.3390/ijms22157920)
Supplement: Supplementary file 1 [file ijms-22-07920-s001.zip › ijms-1263158-supplementary.pdf]

Supplementary Material

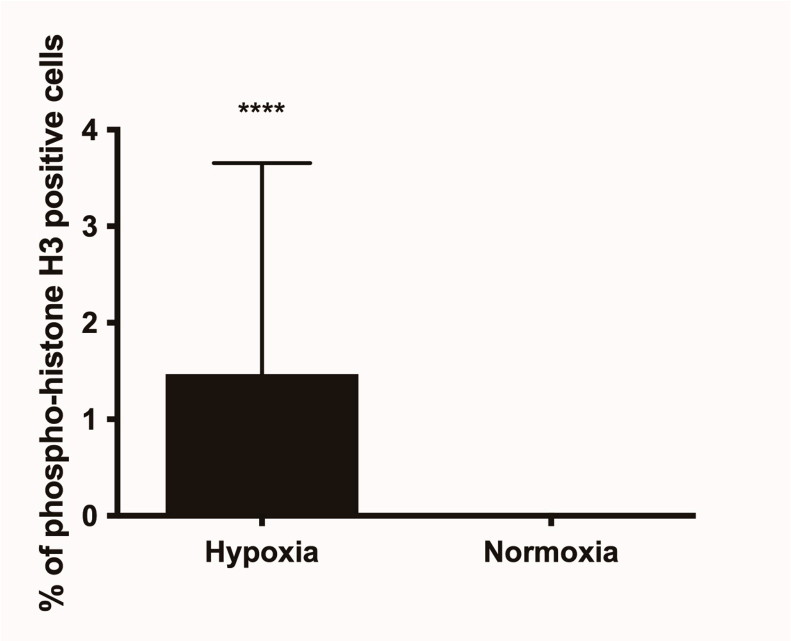

Figure S1. Percentage of phospho-histone H3 positive cells. The percentage of phospho-histone H3 postive cells was zero in the normoxia treated patches. Comparison was performed with non-parametric test (Mann-Whitney-U).

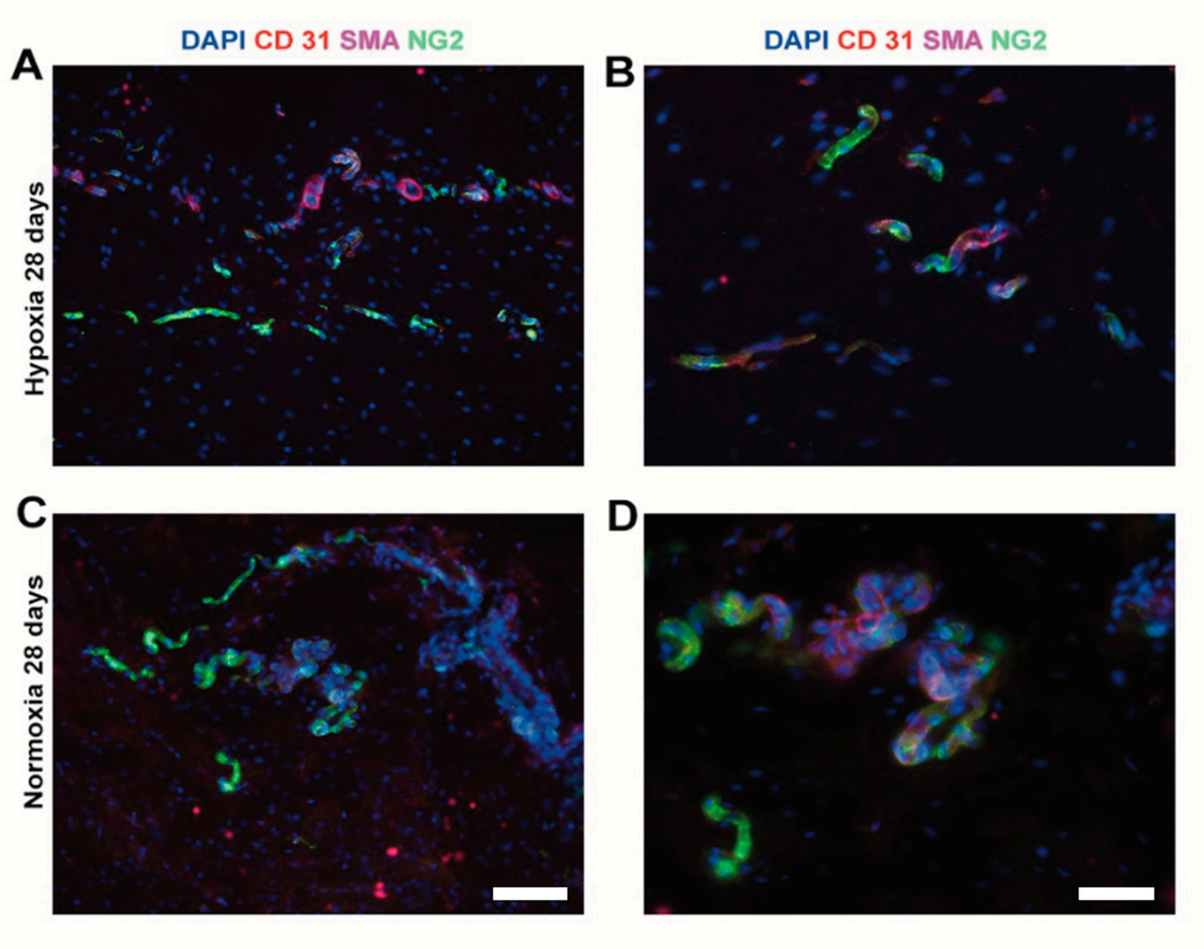

Figure S2. Characterization of vessel morphology: The blood vessel morphology shows a stable structure of capillaries with vessels covered by pericytes (NG2-positve cells) in both experimental group at 28 days following in vivo implantation at low (20x) and high (40x) magnifications (scale bars = 50µm and 25µm, respectively).
